# Supplementary material for: Temporal Trends of Major Bleeding and Its Prediction by the Academic Research Consortium-High Bleeding Risk Criteria in Acute Myocardial Infarction
Source: J Clin Med. 2022 Feb 14;11(4):988. doi: 10.3390/jcm11040988 (PMC8875601; doi:10.3390/jcm11040988)
Supplement: Supplementary file 1 [file jcm-11-00988-s001.zip › jcm-1556439-supplementary.pdf]

Supplementary Materials

Temporal trends of major bleeding and its prediction by the Academic Research Consortium-High Bleeding Risk criteria in acute myocardial infarction

Sungwook Byun, MD, Eun Ho Choo, MD, Gyu-Chul Oh, MD, Sungmin Lim, MD, PhD, Ik Jun Choi, MD, PhD, Kwan Yong Lee, MD, PhD, Su Nam Lee, MD, PhD, Byung-Hee Hwang, MD, Chan Joon Kim, MD, PhD, Mahn-Won Park, MD, PhD, Chul Soo Park, MD, PhD, Hee-Yeol Kim, MD, PhD, Ki-Dong Yoo, MD, PhD, Doo Soo Jeon, MD, PhD, Ho Joong Youn, MD, PhD, Wook Sung Chung, MD, PhD, Min Chul Kim, MD, PhD, Myung Ho Jeong, MD, PhD, Hyeon-Woo Yim, MD, PhD, Youngkeun Ahn, MD, PhD, and Kiyuk Chang, MD, PhD

Supplementary methods. Definition of AMI and inclusion and exclusion criteria in COREA-AMI registry

Table S1. Study definitions of individual ARC-HBR criteria compared with the original ARC-HBR definitions

Table S2. Annual incidence of BARC 3, 5 bleeding within 1 year of PCI in AMI population between 2004 and 2014

Table S3. Composition of types of PCI in AMI population each year

Table S4. Prevalence of bleeding risk factors in AMI population by period

Table S5. Cumulative incidence of primary bleeding outcome from Index PCI to 1 month and from 1 month to 1 year in AMI population

Supplementary methods. Definition of AMI and inclusion and exclusion criteria in COREA-AMI registry

Definition of AMI

Patients were diagnosed with AMI if their concentrations of cardiac biomarkers were at least one unit above the 99th percentile with temporal variations and at least one of the followings was fulfilled: 1) symptoms of ischemia, 2) new significant ST-segment-T wave changes or new left bundle branch block, 3) pathological Q waves, 4) imaging evidence of new loss of viable myocardium or new regional wall motion abnormality, or 5) angiographically detected intracoronary thrombus.

Patients were divided into two groups based on electrocardiographic findings, ST segment elevation myocardial infarction (STEMI) or non-ST segment elevation myocardial infarction (NSTEMI). Patients assigned to the STEMI group demonstrated typical ST segment elevation in two contiguous leads or ST-segment elevation in lead aVR or atypical presentations, such as bundle branch block. All other patients were assigned to the NSTEMI group.

Inclusion and exclusion criteria in COREA-AMI registry

In the COREA-AMI registry, patients over 19 years of age who were diagnosed with AMI and treated with PCI using stents were included. AMI patients who were only treated by conservative strategy were excluded.

Table S1. Study definitions of individual ARC-HBR criteria compared with the original ARC-HBR definitions.

ARC-HBR criteria	Study definition	Original ARC-HBR definition [1]
**Major criteria**		
**Anticoagulation**	Oral anticoagulation (VKA or NOAC) at discharge and follow-up	Anticipated use of long-term oral anticoagulation
**Severe CKD**	No difference	eGFR <30 mL/min
**Severe anemia**	No difference	Hemoglobin <11 g/dL
**Thrombocytopenia**	No difference	Platelet count <100 x10^9/L
**Malignancy**	Any active or non-active malignancy	Active malignancy within the past 12 months
**Prior bleeding**	Prior any spontaneous bleeding	Spontaneous bleeding in the past 6 months or at any time, if recurrent
**Liver disease**	Liver disease with thrombocytopenia	Liver cirrhosis with portal hypertension
**Prior ICH**	Previous spontaneous or traumatic ICH	Previous spontaneous ICH; traumatic ICH within the past 12 months; presence of bAVM; Moderate or severe ischemic stroke within the past 6 months
	Not available	Chronic bleeding diathesis
	Not available	Non-deferrable major surgery on dual antiplatelet therapy
	Not available	Recent major surgery or major trauma within 30 days before PCI
**Minor criteria**		
**Age >75**	No difference	Age ≥75 years
**Moderate CKD**	No difference	eGFR 30–59 mL/min

**Moderate anemia**	No difference	Hemoglobin 11–12.9 g/dL for men and 11–11.9 g/dL for women
**NSAID**	NSAID use at discharge and follow-up	Long-term use of oral NSAIDs or steroids
**Prior any stroke**	Prior any stroke	Any ischemic stroke at any time not meeting the major criterion
	Not available	Spontaneous bleeding within the past 12 months not meeting the major criterion

ARC-HBR, Academic Research Consortium for High Bleeding Risk; bAVM, brain arteriovenous malformation; CKD, chronic kidney disease; COREA-AMI, CardiOvascular Risk and idEntificAtion of potential high-risk population in Acute Myocardial Infarction; eGFR, estimated glomerular filtration rate; ICH, intracranial hemorrhage; NOAC, non-vitamin K antagonist oral anticoagulants; NSAIDs, nonsteroidal anti-inflammatory drugs; PCI, percutaneous coronary intervention; VKA, vitamin K antagonist.

Table S2. Annual incidence of BARC 3, 5 bleeding within 1 year of PCI in AMI population between 2004 and 2014.

Year	No. of AMI patients with BARC 3, 5 bleeding within 1 year of PCI	No. of AMI patients without BARC 3, 5 bleeding within 1 year of PCI	Incidence (%)
**2004**	2	112	1.8
**2005**	8	285	2.7
**2006**	25	471	5.0
**2007**	12	499	2.3
**2008**	40	828	4.6
**2009**	57	974	5.5
**2010**	74	1252	5.6
**2011**	38	1314	2.8
**2012**	83	1479	5.3
**2013**	125	1461	7.9
**2014**	67	1085	5.8

AMI, acute myocardial infarction; BARC, Bleeding Academic Research Consortium; PCI, percutaneous coronary intervention.

Table S3. Composition of types of PCI in AMI population each year.

Year	Total PCl	PCI with 1st DES (%)	PCI with 2nd DES (%)	*Other types of PCI (%)
**2004**	114	105 (92.1)	0 (0)	9 (7.9)
**2005**	293	280 (95.6)	1 (0.3)	12 (4.1)
**2006**	496	446 (89.9)	30 (6.0)	20 (4.0)
**2007**	511	437 (85.5)	44 (8.6)	30 (5.9)
**2008**	868	540 (62.2)	165 (19.0)	163 (18.8)
**2009**	1031	402 (39.0)	478 (46.4)	151 (14.6)
**2010**	1326	157 (11.8)	1001 (75.5)	168 (12.7)
**2011**	1352	51 (3.8)	1020 (75.4)	281 (20.8)
**2012**	1562	6 (0.4)	1357 (86.9)	199 (12.7)
**2013**	1586	1 (0.1)	1401 (88.3)	184 (11.6)
**2014**	1152	0 (0)	1024 (88.9)	128 (11.1)

* Other types of PCI include thrombus aspiration only, balloon angioplasty, bare-metal stent and biodegradable stent

AMI, acute myocardial infarction; DES, drug eluting stent; PCI, percutaneous coronary intervention.

Table S4. Prevalence of bleeding risk factors in AMI population by period.

*Bleeding risk factor (%)	Total (n=10,291)	2004–2008 (n=2,282)	2009–2014 (n=8,009)	*P* for trend
**Age >75 (minor)**	2315 (22.5)	391 (17.1)	1924 (24.0)	<.001
**Moderate anemia (minor)**	1970 (19.1)	403 (17.7)	1567 (19.6)	0.04
**Moderate CKD (minor)**	1915 (18.6)	409 (17.9)	1506 (18.8)	0.35
**Severe anemia (major)**	1127 (11.0)	274 (12.0)	853 (10.7)	0.07
**Severe CKD (major)**	688 (6.7)	142 (6.2)	546 (6.8)	0.34
**Prior any stroke (minor)**	623 (6.1)	113 (5.0)	510 (6.4)	0.01
**Malignancy (major)**	348 (3.4)	63 (2.8)	285 (3.6)	0.07
**Anticoagulation (major)**	336 (3.3)	48 (2.1)	288 (3.6)	0.001
**Prior ICH (major)**	127 (1.2)	23 (1.0)	104 (1.3)	0.32
**Thrombocytopenia (major)**	113 (1.1)	28 (1.2)	85 (1.1)	0.58
**NSAID (minor)**	88 (0.9)	10 (0.4)	78 (1.0)	0.02
**Prior bleeding (major)**	40 (0.4)	10 (0.4)	30 (0.4)	0.81
**Liver disease (major)**	17 (0.2)	5 (0.2)	12 (0.1)	0.67

* The individual ARC-HBR criteria were used as bleeding risk factors, marked as major or minor.

AMI, Acute Myocardial Infarction; ARC-HBR, Academic Research Consortium for High Bleeding Risk; CKD, chronic kidney disease; ICH, intracranial hemorrhage; NSAIDs, nonsteroidal anti-inflammatory drugs.

Table S5. Cumulative incidence of primary bleeding outcome from Index PCI to 1 month and from 1 month to 1 year in AMI population

	**ARC-defined HBR (n=3371)**		**ARC-defined non-HBR (n=6920)**		**Cox analysis**	
	**Events / Patients at risk**	**Incidence rate (%)**	**Events / Patients at risk**	**Incidence rate (%)**	**Hazard ratio (95% CI)**	***P* value**
Index PCI to 1 month	221 / 3371	6.6	156 / 6920	2.3	3.08 (2.51–3.78)	<.001
1 month to 1 year	108 / 2779	3.9	46 / 6531	0.7	5.91 (4.19–8.35)	<.001

AMI, acute myocardial infarction; ARC, Academic Research Consortium; CI, confidence interval; HBR, high bleeding risk; PCI, percutaneous coronary intervention.

Reference

[1] P. Urban, R. Mehran, R. Colleran, D.J. Angiolillo, R.A. Byrne, D. Capodanno, et al., Defining High Bleeding Risk in Patients Undergoing Percutaneous Coronary Intervention, Circulation 140 (2019) 240-261. https://doi.org/10.1161/CIRCULATIONAHA.119.040167.
